# Mechanical thrombectomy in cancer-associated pulmonary embolism: reassuring safety, unclear benefit

**DOI:** 10.1016/j.rpth.2026.106922

**Published:** 2026-08-27

**Authors:** Stanislav Henkin, Gregory Piazza

**Affiliations:** 1 Gonda Vascular Center, Mayo Clinic, Rochester, Minnesota, USA; 2 Thrombosis Research Group, Brigham and Women’s Hospital, Harvard Medical School, Boston, Massachusetts, USA; 3 Division of Cardiovascular Medicine, Brigham and Women’s Hospital, Boston, Massachusetts, USA

*“Just when they think they got the answers, I change the questions.”—Roderick George Toombs*

As the world population grows and ages, cancer cases worldwide are rising steeply [1]. Fortunately, due to earlier detection and improved treatment, mortality due to cancer has been decreasing over the last 30 years [2]. Nevertheless, cancer is still the second leading cause of death worldwide, only behind cardiovascular disease [1]. Venous thromboembolism, encompassing deep vein thrombosis and pulmonary embolism (PE), is a common complication of cancer, and in some cases, its therapy. In fact, compared to the general population, individuals with cancer demonstrate a greater than 10-fold increased risk for venous thromboembolism; an even greater risk (>20-fold) has been estimated for those who are receiving chemotherapy [3]. Despite a decrease in cancer-related mortality, PE-related mortality in patients with cancer has increased by >40% over the last 10 years [4]. Indeed, acute PE is the second leading cause of death in patients with active cancer [4]. Posing an additional challenge to clinician and patient, treatment of PE in the setting of cancer is complicated by both a higher risk of recurrent events as well as bleeding [5]. Accordingly, there is a profound need for improved therapeutic strategies for acute PE in this vulnerable patient population to improve both short- and long-term outcomes.

While anticoagulation is the cornerstone of acute PE management in all patients including those with malignancy [6], the role of advanced strategies, such as systemic thrombolysis, catheter-directed thrombolysis (CDT), and mechanical thrombectomy (MT), is less clear in those with cancer. Reperfusion therapy is typically considered in patients with intermediate-high and high-risk PE (encompassing American Heart Association/American College of Cardiology PE clinical categories C3-E2) [[7], [8], [9], [10]]. Over the past decade, catheter-based therapies have garnered great clinical and research interest because of the reduced risk of major bleeding, especially intracranial hemorrhage, when compared with systemic fibrinolysis [11]. Contemporary randomized controlled trial data—using very low doses of fibrinolytic drug in catheter-based fibrinolysis [9] and avoiding use of MT altogether [12]—appear to have answered a very important clinical question: can reperfusion therapy for PE be accomplished while minimizing the risk of intracranial hemorrhage? The most recent evidence-based clinical practice guidelines recognize the growing importance of catheter-based therapy in the treatment of more severe presentations of PE [6].

However, the accelerating acquisition of new randomized data focused on catheter-based therapy for acute PE generates many new questions to replace the ones it answers. Importantly, questions abound regarding specific patient populations, such as the advanced elderly, those at the extremes of body mass index, and patients with active cancer. Randomized controlled trials of catheter-based therapy have either excluded such patients or enrolled so few that subgroup analyses are too underpowered to reliably inform on efficacy and safety. Patients with cancer have been notably excluded from or understudied in randomized trials of advanced therapies for acute PE. Accordingly, the National Comprehensive Cancer Network Guidelines reserve advanced therapies for hemodynamically unstable patients or those who deteriorate despite therapeutic anticoagulation [13]. The lack of data precluding a stronger recommendation has consequently limited the frontline clinician caring for patients with more severe manifestations of PE in the setting of cancer. In a National Inpatient Sample of >1 million patients hospitalized with acute PE (20% with active cancer), compared with patients with acute PE but without active cancer, patients with acute PE and active cancer were significantly less likely to receive advanced therapies, including systemic thrombolysis, CDT, or MT [14]. In this population, presence of active cancer, especially metastatic cancer, was associated with significant increase in all-cause mortality during hospitalization [14]. The logical question arises—would outcomes be different if patients with cancer were to receive advanced therapies for acute PE management?

In this multicenter observational study across 8 centers in Poland, Banaszkiewicz et al. [15] evaluated outcomes of patients with intermediate- and high-risk PE (categories C3-E2), with and without active cancer, and contraindication to full-dose systemic thrombolysis due to perceived high risk of bleeding, who underwent MT using Penumbra device. The study population included 290 patients; 18% (53 patients) had active cancer and the majority (∼70%) were receiving anticancer therapies at the time of acute PE. The noncancer and cancer populations were similar overall, although patients without cancer were significantly more likely to present with normotensive shock based on reduced cardiac index (34% vs 13%, *P* = .003). One in 5 patients in both groups required catecholamines for hemodynamic support, consistent with American Heart Association/American College of Cardiology clinical category E1 PE. In both groups, low molecular weight heparin (∼60%) was the most common initial anticoagulation strategy, consistent with recent guidelines recommendations [6]. Risk of access site complications (<3%) were low in both groups, while procedural blood loss was similar (∼330 mL). With MT, hemodynamics (ie, mean pulmonary arterial pressure) and imaging characteristics (right ventricular-to-left ventricular ratio) significantly improved in both groups, without observed intragroup differences. Importantly, in-hospital mortality (6.4% vs 9.4%), likely more reflective of PE-related mortality, and major bleeding (0.8% vs 1.9%) were statistically similar between noncancer and cancer patients. Still, compared to patients without cancer, 30-day all-cause mortality was significantly higher in patients with cancer (17% vs 6.8%, *P* = .027).

The study by Banaszkiewicz et al. [15] fills an important gap in real-world data with respect to short-term outcomes in patients with cancer-associated PE undergoing MT [15]. Safety and efficacy of MT in patients with cancer-associated PE appear to be similar compared with those in patients without cancer. However, it is important to note that this study was likely underpowered to detect statistically significant differences in outcomes as <20% of the study population had active cancer. Unfortunately, 30-day mortality in patients with cancer remains high, likely reflective of underlying morbidity associated with advanced cancer rather than PE-related sequelae. Additionally, this study did not evaluate outcomes in patients who underwent MT compared with those who were managed conservatively with anticoagulation alone. Still, MT is an attractive option for patients with PE and perceived high risk of bleeding as MT avoids the need for systemic or local thrombolytic. In a Pulmonary Embolism Response Team Registry of ∼3000 patients, 19.3% had active malignancy and were significantly more likely to undergo MT compared to CDT or systemic thrombolysis [16]. In a prior retrospective observational study of patients with cancer-associated PE, compared with anticoagulation alone, catheter-based therapy, including CDT and MT, was associated with 11% relative risk reduction in in-hospital mortality and 25% relative risk reduction in 90-day readmission without an increased risk of major bleeding [17]. Outcomes between CDT and MT were similar [17]. While cohort studies including the current analysis support the feasibility and relative safety of MT for PE in patients with cancer, we are left with a number of questions that require more randomized data. For example, in light of rapid adoption of catheter-based options, the question is no longer “Can we feasibly perform catheter-based intervention for PE patients with cancer?” but rather “Does catheter-based therapy for PE provide clinical benefit with an adequate safety margin in patients with cancer compared with standard of care anticoagulation?” Furthermore, the goal of future studies should not be to show similar safety and efficacy in patients with PE in the setting of cancer compared with those without cancer, but to understand the subset of this population that would receive a net clinical benefit from intervention and when to select a particular device in a particular patient.

As the number of cancer cases worldwide increases, these real-world data provide foundational evidence that the medical community needs to plan appropriately-powered randomized trials to fully understand the safety and efficacy of advanced therapies, be it MT or CDT, in patients with cancer-associated PE. While awaiting such studies, the current study reassures us that the risk of major bleeding in patients with cancer-associated PE does not seem to be significantly different than those without cancer. That said, the divergence between similar in-hospital outcomes but significantly higher 30-day mortality in patients with cancer-associated PE is profound. This distinction is important for trial design as future trials should incorporate cancer type, tumor burden, and anticipated oncologic prognosis to understand which patients may most benefit from either CDT or MT and to distinguish between PE-related morbidity and cancer-related morbidity. In the careful preparation of such trials, we will not only position ourselves to answer pressing questions in this vulnerable patient population, but we will better prepare ourselves for the next round of questions challenging our clinical care.
